# Supplementary material for: Spatial patterning of chloroplasts and stomata in developing cacao leaves
Source: Commun Biol. 2025 Apr 4;8:554. doi: 10.1038/s42003-025-08019-6 (PMC11968909; doi:10.1038/s42003-025-08019-6)
Supplement: Supplementary file 2 — Supplemental Information [file 42003_2025_8019_MOESM2_ESM.pdf]

Article title: Spatial patterning of chloroplasts and stomata in developing cacao leaves

Authors: Insuck Baek[1], Seunghyun Lim[2], Visna Weerarathne[3], Dongho Lee[4], Jacob Botkin[5], Silvas Kirubakaran[3], Sunchung Park[2], Moon S. Kim[1], Lyndel W. Meinhardt[2], Ezekiel Ahn[2,*]

The following Supporting Information is available for this article:
Fig. S1 Pearson's correlation analysis of chloroplast cluster variation patterns.
Fig. S2 Spatial variation of chloroplast cluster morphology traits in Stage C cacao leaves.
Fig. S3 Spatial variation of chloroplast clusters and stomatal size.
Fig. S4 Spatial variation of stomatal morphology traits in Stage C cacao leaves.
Fig. S5 Detailed comparison of SVM model performance for stomatal classification using all seven and top three morphological features.
Fig. S6 Detailed comparison of SVM model performance for chloroplast classification. .

Fig. S1: Pearson's correlation analysis of chloroplast cluster variation patterns.

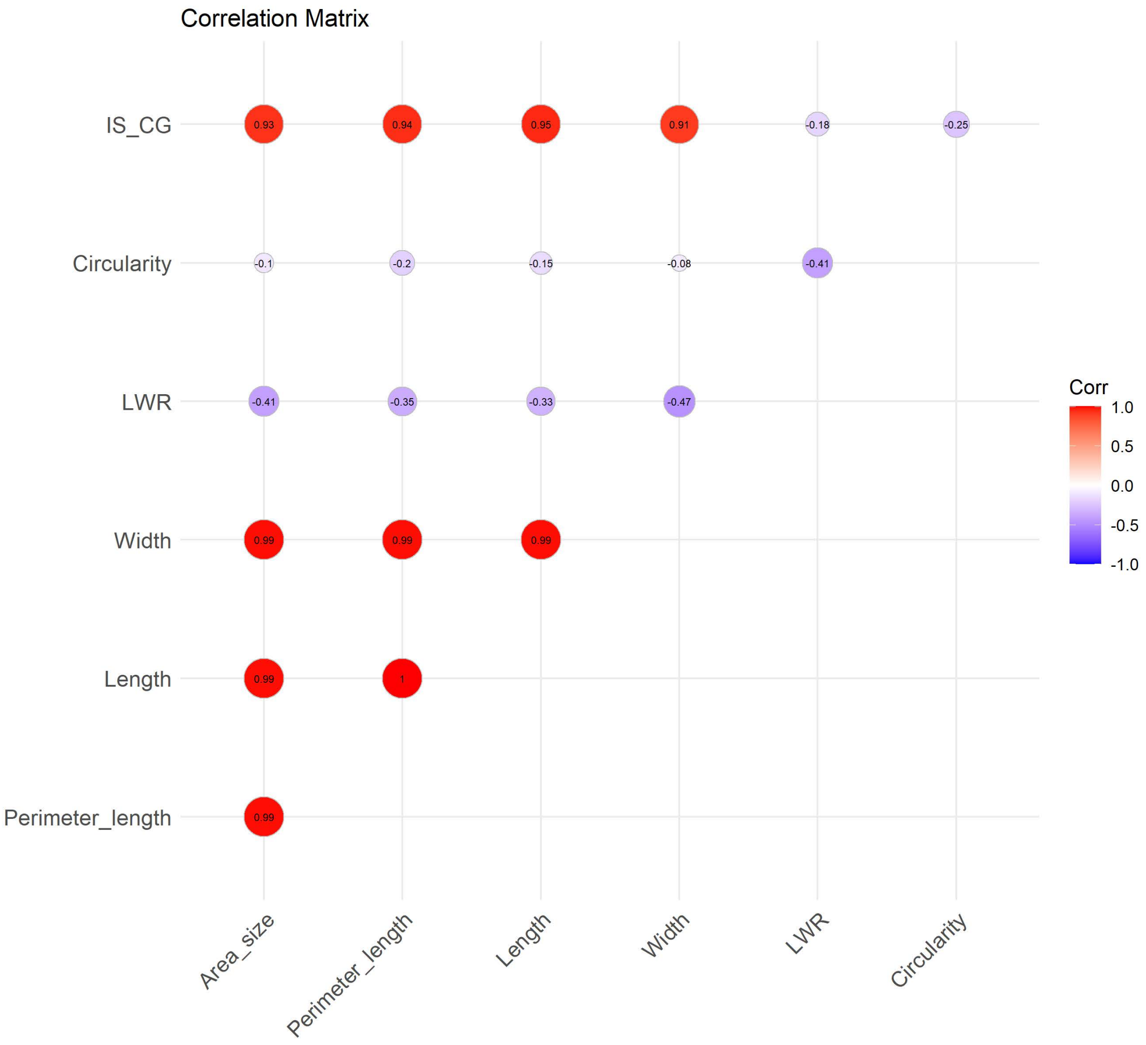

This Fig. presents scatter plots and significance circles depicting correlations between various chloroplast variation traits observed under a 10X microscope. Pearson's correlation analysis reveals statistically significant correlations ($p < 0.0001$) for most trait pairs, except for LWR-area size and circularity-width.

Fig. S2: Spatial variation of chloroplast cluster morphology traits in Stage C cacao leaves.

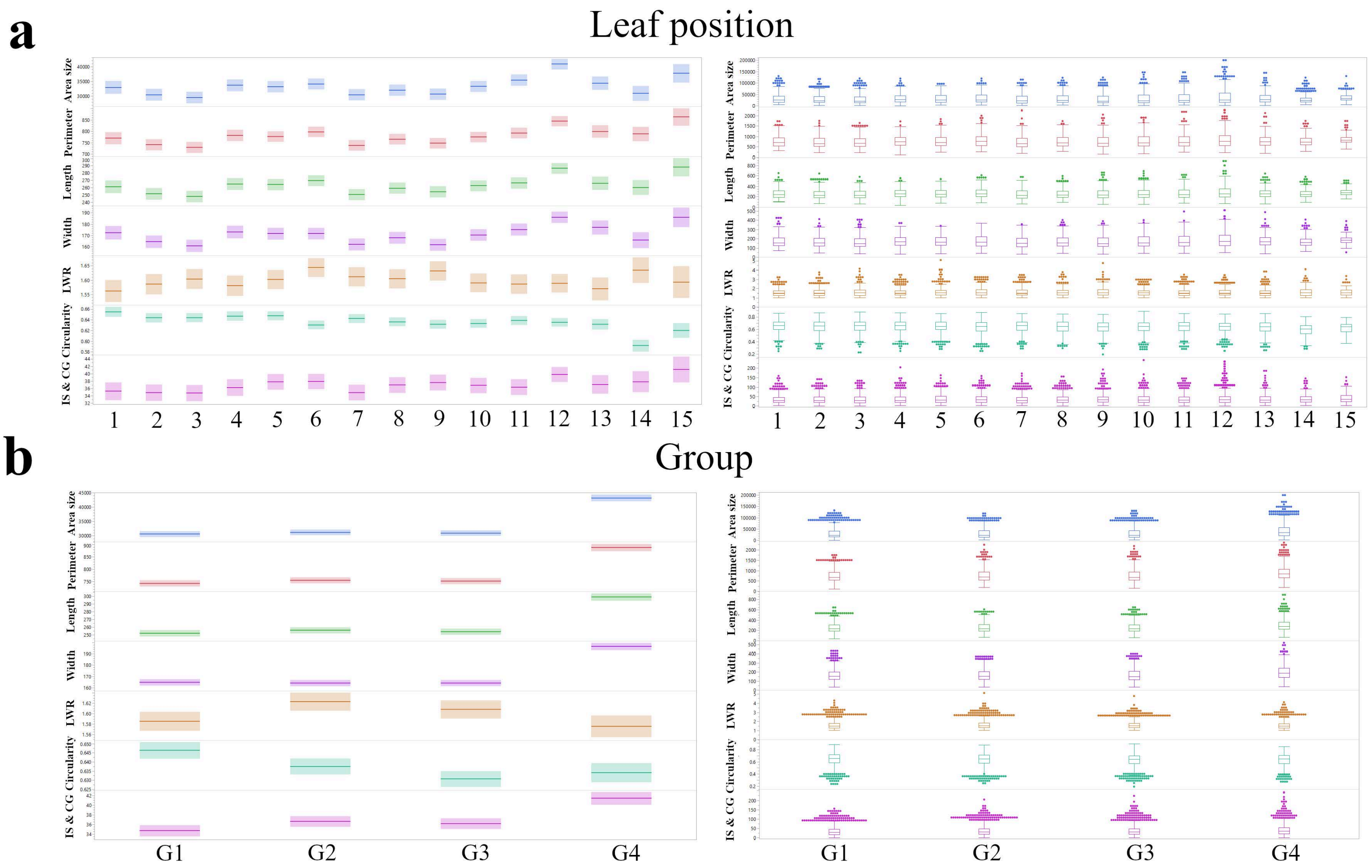

This figure displays the mean (horizontal line within each bar) and 95% confidence intervals (vertical bars) for seven chloroplast cluster morphology traits: area size (μm²), perimeter (μm), length (μm), width (μm), length-to-width ratio (LWR, unitless), circularity (0-100), and distance between the intersection of length and width and the center of gravity (IS & CG, μm). Data are shown for individual leaf positions (1-15) (a) and grouped leaf regions (G1-G4) (b). On the right side, box plots provide a detailed visualization of the identical data, showing median, quartiles, and outliers. Overlapping confidence intervals suggest a lack of statistically significant difference between the means of those groups.

Fig. S3: Spatial variation of chloroplast clusters and stomatal size.

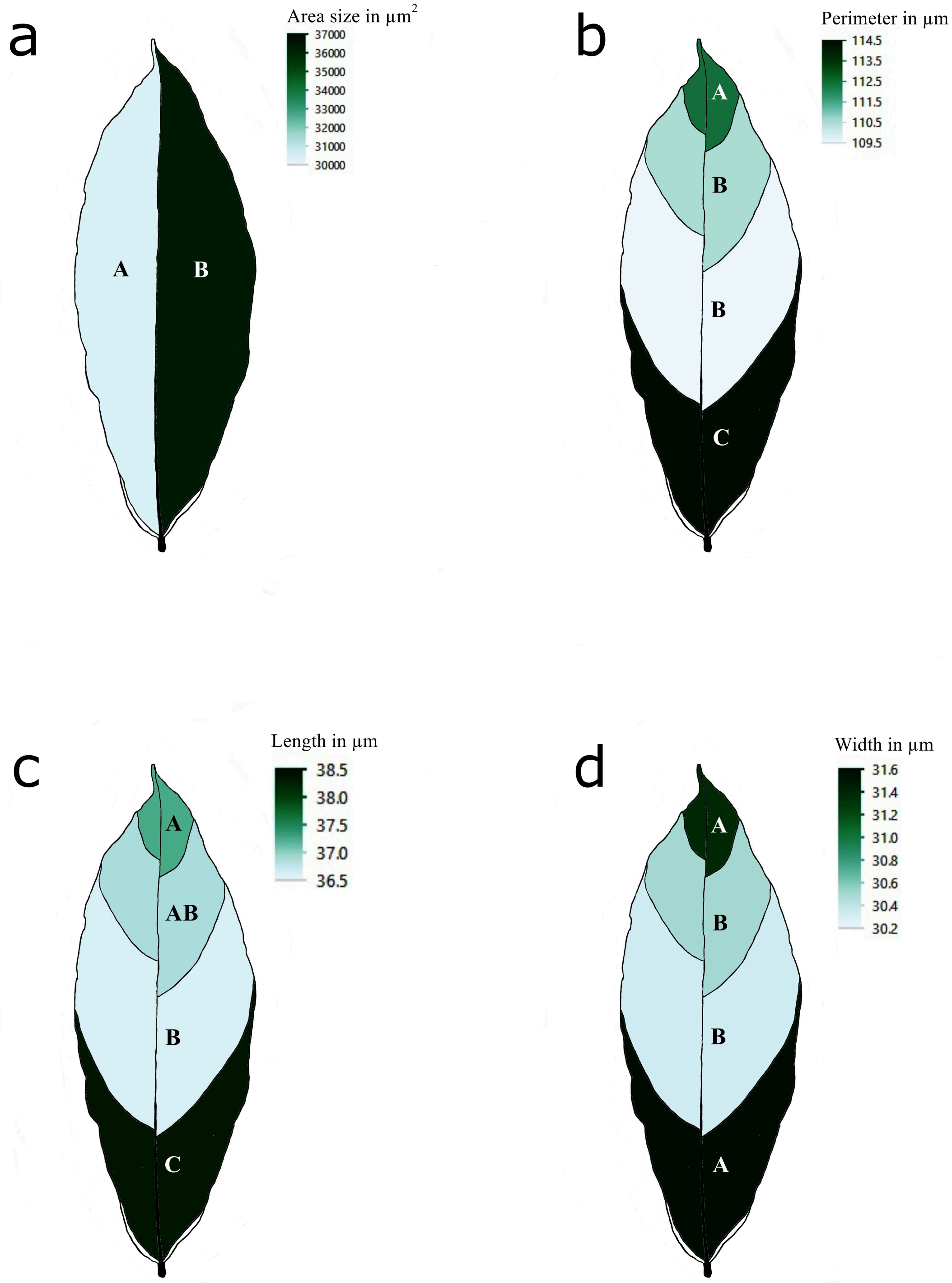

(a) Chloroplast cluster area variation between left and right leaf halves. (b-d) Stomatal size (perimeter, length, and width) variation across G1-4 regions. Greener tones indicate larger values. Different letters denote significant differences between groups (t-test for (a), Tukey's HSD for (b-d)). n = 7,652 for chloroplast clusters; n = 11,809 for stomata.

Fig. S4: Spatial variation of stomatal morphology traits in Stage C cacao leaves.

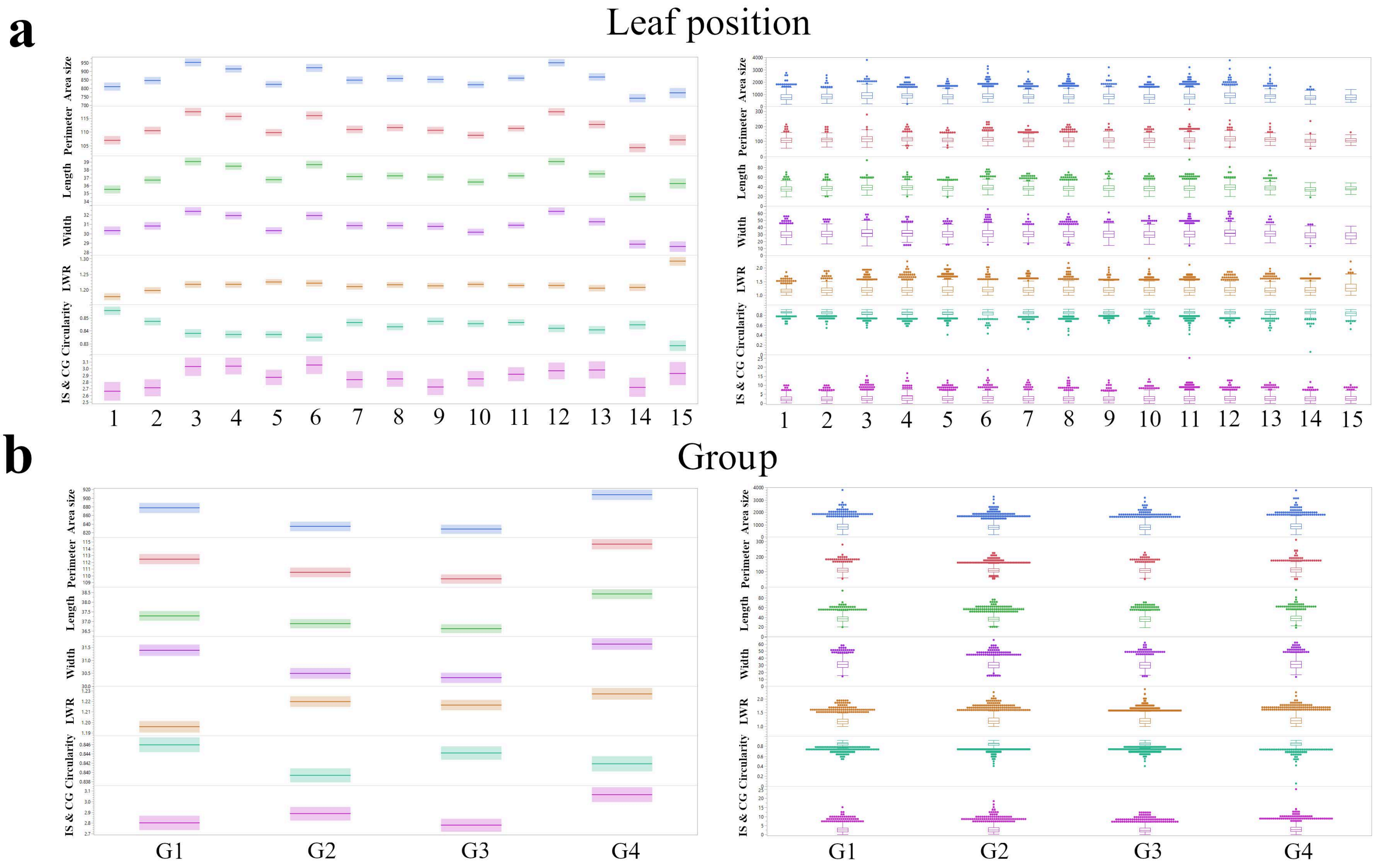

This figure displays the mean (horizontal line within each bar) and 95% confidence intervals (vertical bars) for seven stomatal morphology traits: area size (µm²), perimeter (µm), length (µm), width (µm), length-to-width ratio (LWR, unitless), circularity (0-100), and distance between the intersection of length and width and the center of gravity (IS & CG, µm). Data are shown for individual leaf positions (1-15) (a) and grouped leaf regions (G1-G4) (b). On the right side, box plots provide a detailed visualization of the identical data, showing median, quartiles, and outliers. Overlapping confidence intervals suggest a lack of statistically significant difference between the means of those groups.

Fig. S5: Detailed comparison of SVM model performance for stomatal classification using all seven and top three morphological features.

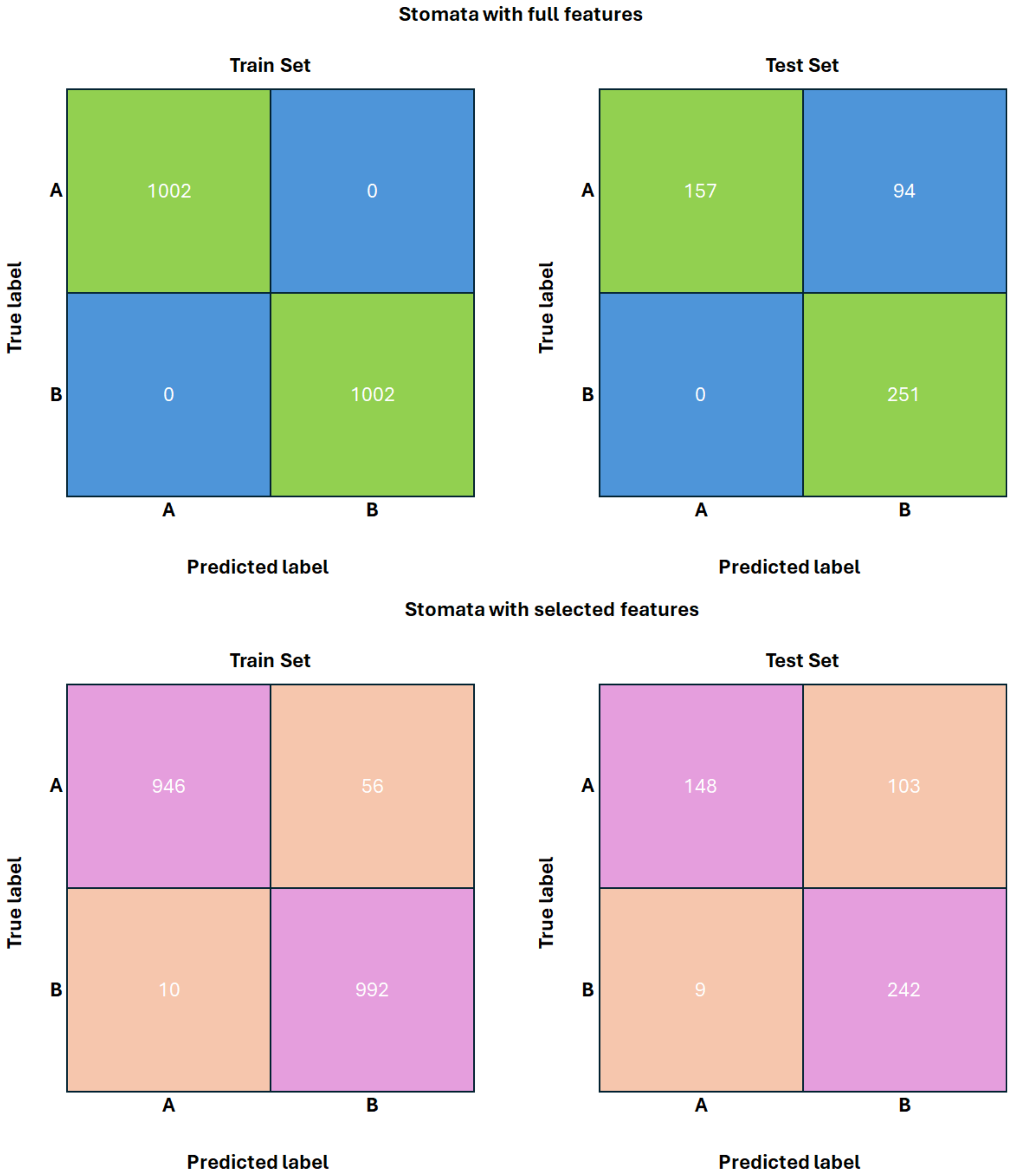

This Fig. details the performance of the SVM model for stomatal classification using two feature sets: all seven morphological features and the top three features selected by ANOVA. High accuracy was achieved for both feature sets in the training dataset. Notably, stomata from the basal G4 leaf region (Group B) exhibited higher classification accuracy compared to those from other G1-3 leaf regions (Group A).

Fig. S6: Detailed comparison of SVM model performance for chloroplast classification.

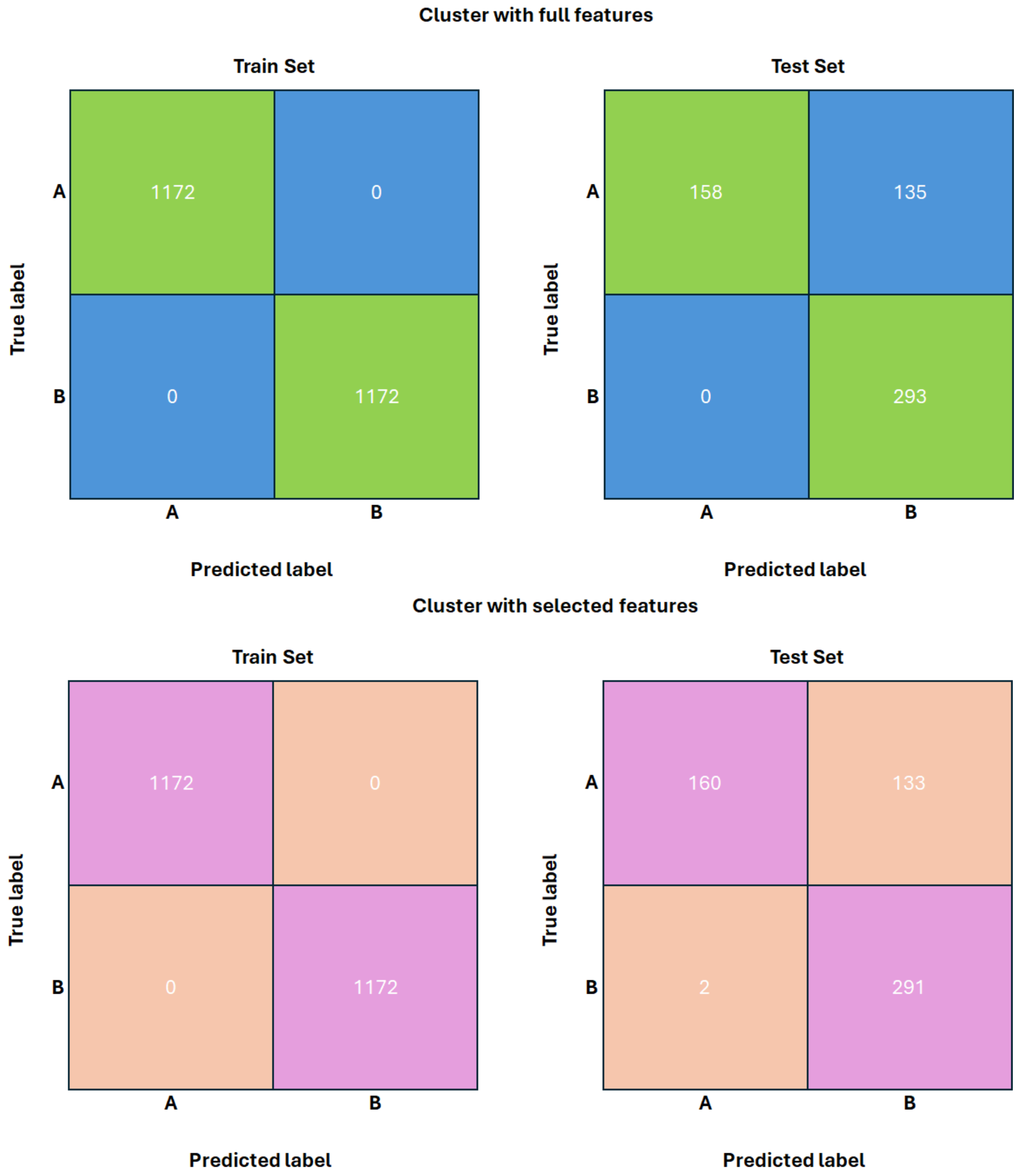

This Fig. provides a detailed comparison of SVM model performance for chloroplast classification using two feature sets: all seven morphological features and the top three features. Like stomata classification, high accuracy was achieved for both feature sets in the training dataset. Notably, chloroplast clusters from the basal G4 leaf region (Group B) showed higher classification accuracy than those from the other G1-3 leaf regions (Group A).
