# Supplementary material for: Spatial patterning of chloroplasts and stomata in developing cacao leaves
Source: Commun Biol. 2025 Apr 4;8:554. doi: 10.1038/s42003-025-08019-6 (PMC11968909; doi:10.1038/s42003-025-08019-6)
Supplement: Supplementary file 3 — Description of Additional Supplementary Files [file 42003_2025_8019_MOESM3_ESM.docx]

Description of Additional Supplementary Files

**File Name:** Supplementary Data 1

**Description:** All raw phenotypic data.

**File Name:** Supplementary Data 2

**Description:** List of leaf, chloroplast, and stomatal traits measured in the study, including their abbreviations, descriptions, and units.
